# Latest Insights into the In Vivo Studies in Murine Regarding the Role of TRP Channels in Wound Healing—A Review

**DOI:** 10.3390/ijms25126753

**Published:** 2024-06-19

**Authors:** Alexandra Grigore, Oana Andreia Coman, Horia Păunescu, Mihnea Costescu, Ion Fulga

**Affiliations:** Department of Pharmacology and Pharmacotherapy, Faculty of Medicine, “Carol Davila” University of Medicine and Pharmacy, 050474 Bucureșt, Romania; alexandra_grigore@drd.umfcd.ro (A.G.); oana.coman@umfcd.ro (O.A.C.); mihnea.costescu@umfcd.ro (M.C.); ion.fulga@umfcd.ro (I.F.)

**Keywords:** wound healing, transient receptor potential (TRP) channels, ion channels

## Abstract

Wound healing involves physical, chemical and immunological processes. Transient receptor potential (TRP) and other ion channels are implicated in epidermal re-epithelization. Ion movement across ion channels can induce transmembrane potential that leads to transepithelial potential (TEP) changes. TEP is present in epidermis surrounding the lesion decreases and induces an endogenous direct current generating an epithelial electric field (EF) that could be implicated in wound re-epithelialization. TRP channels are involved in the activation of immune cells during mainly the inflammatory phase of wound healing. The aim of the study was to review the mechanisms of ion channel involvement in wound healing in in vivo experiments in murine (mice, rats) and how can this process be influenced. This review used the latest results published in scientific journals over the last year and this year to date (1 January 2023–31 December 3000) in order to include the in-press articles. Some types of TRP channels, such as TRPV1, TRPV3 and TRPA1, are expressed in immune cells and can be activated by inflammatory mediators. The most beneficial effects in wound healing are produced using agonists of TRPV1, TRPV4 and TRPA1 channels or by inhibiting with antagonists, antisense oligonucleotides or knocking down TRPV3 and TRPM8 channels.

## 1. Introduction

The tegument is an important organ of the body, capable of performing various vital functions. It can take various forms, being elastic and has the role of communication between the brain and the environment [1].

The skin accounts for 10–16% of the weight of an adult and consists of the following three layers: the epidermis (outer layer), the dermis and the subcutaneous tissue (hypodermis). Melanocytes, Langerhans cells and Merkel cells are present in the stratified squamous epithelium of keratinocytes that make up the epidermis, the outermost layer. Dermis, known for providing structural integrity, elasticity and nutrition, is essentially a connective tissue that is composed of fibroblasts and an extracellular matrix that is rich in collagen and elastic fibers [2,3,4], and also contains blood and lymphatic vessels, sebaceous glands, sweat glands, nerve endings and hair follicles invaginated from the epidermis [1,5]. The hypodermis lies in the deepest part of the dermis and is also known as the subcutaneous fascia. Adipose lobules and certain skin appendages, including hair follicles, sensory neurons and blood vessels, are found in the deepest layer of skin [6].

The repair of tissue that loses the integrity is facilitated through the interaction of tissue-resident immune, stromal and epithelial cells, and infiltrating immune cells, in a complex process [7].

Hemostasis is initiated by injury to the vascular endothelium and concludes by the formation and degradation of a fibrin clot that lasts for minutes. After hemostasis, the inflammatory phase starts and lasts for 3–5 days [8]. This phase is marked by the recruitment of immune cells, reduction in vasoconstriction and increase in vessel permeability due to local tissue hypoxia and acidosis [1,9].

During the inflammatory phase, growth factor signaling triggers the proliferation stage, which may last for up to 2 weeks. Epithelization of the wound surface, the formation of new granulation tissue and angiogenesis are signs of this process, which fills some of the defects left behind by injuries [10,11,12,13].

Remodeling is the third and final stage, and it can last for 1–2 years in humans. The process of remodeling is accompanied by fibroblast proliferation and involves degradation and reorganization of the extracellular matrix (ECM), blood vessels and granulation tissue. Then, a scar is formed from organized collagen that has a weaker (50–80%) share stress resistance than normal tissue [14,15].

Despite the recent progress in this domain, no current experimental models fully predict the outcomes of clinical trials [16,17]. The complexity of the healing process is not recreated by in vitro models, even though they focus on repair pathways of specific cell populations [15,18]. To understand the physiological and pathological mechanisms of tissue repair, animal models are essential [16,17,18,19]. Even though there are differences between species and strains, the murine models are good instruments in understanding normal and pathological cutaneous repair.

The dermis and epidermis in human and murine skin have considerably different thicknesses and numbers of layers. While human skin is generally thick (over 100 mm), firm and firmly attached to the underlying tissues, murine skin is thinner (less than 25 mm) and it is loose [18,19,20]. Murine epidermis has only 2 or 3 layers, which decreases its barrier function and enhances percutaneous absorption, while human epidermis is composed of 5 to 10 cell layers [21,22,23]. The thickness of epidermis, dermis and subcutaneous tissue differs depending on site, age, sex and nutrition, both in mice and humans. Mice males have a thicker and 40% firmer dermis than females. Coversely, mice females have a thicker epidermis and subcutaneous tissue than males [18]. When analyzing preclinical studies, it is important to take these facts into account as they affect the biomechanics of healing.

The panniculus carnosus consists of a thin layer of striated muscle that is intimately attached to the skin and fascia of most mammals. Skin biomechanics are affected by panniculus carnosus, which can be found in the subcutaneous tissue of rodents but is rare in humans [20]. The skin’s potential for contraction is greatly enhanced by a thin layer of muscle tissue, and large wounds heal mainly through contraction and union of the borders of wound. Up to 90% of excisional wounds in mice are closed through contraction. Contrary to mice, the human dermis is tightly connected to the subcutaneous tissues, and the contraction is highly variable and much less pronounced [17].

The healing process for skin wounds generally involves the activation of granulation tissue, fibroblasts, myofibroblasts, endothelial vascular cells and epithelial cells, internally and externally, through mechanical stimuli. Myofibroblast forces cause the contraction of the cutaneous wound, which is further exacerbated by external forces such as scratching, compression and skin tension [24]. To control the volume of ECM, collagen and fibronectin are produced by fibroblasts and collagenase is utilized [25]. Proteins within the ECM are synthesized and broken down sequentially, resulting in the reshaping of three-dimensional ECM structures. The binding of cells to matrix proteins can cause cells to deform due to the small forces exerted [26]. Deformed cells have different potential and functionality to ion channels, and are possibly sensitive to mechanical forces.

The process of wound healing also involves an overactive immune system. The immune response to wound healing is heavily dependent on keratinocytes, endothelial cells, fibroblasts, dendritic cells (DCs), neutrophils, monocytes, macrophages and innate lymphocytes including natural killer (NK) cells, γδT cells and skin-resident T lymphocytes. In order to initiate and regulate inflammation, they release significant amounts of cytokines [27]. Pathophysiological signals of lesional process with some features are triggered by environmental factors, and some by changes in local host environment including the ion channels functions.

Ion channels, which are transmembrane proteins, play a significant role in many cellular processes, such as regulating membrane potential, intracellular signaling and cellution [28,29,30], and muscle contraction [31,32].

The mechanical properties of cells that could be involved in covering the area of the wound are described in in vivo and in vitro studies [33].

The movement of individual cells toward the wound center is coordinated by their role to repair small or large barrier defects during wound healing [33,34]. One of the most significant processes for restoring the skin barrier is the migration of epithelial cells into a continuous lamina structure.

Research has focused on the process of initiating multicellular and tissue-level movement upon injury, coordinating during healing, and stopping when wounds heal. When the skin is injured, the epithelial barrier becomes damaged, and ion channels are responsible for generating endogenous electric fields (EFs), which are kept stable by cell junctions [34,35]. Except the mechanical properties of the cells involved in wound healing, the electric properties of these cells or of their microenvironment could be influenced by drugs or appliance of some electric fields from outside. Wound healing also involves calcium ions. Calcium ions are necessary for the differentiation and formation of keratinocytes. To cope with the different calcium needs of keratinocytes (low calcium concentrations for proliferation, high calcium concentrations for differentiation), the epidermis builds up a calcium gradient. Calcium can enter cells either through calcium channels that are specific, or by being released from intracellular stores, specifically from the endoplasmic reticulum [36,37].

K^+^ channels can play a fundamental role in cell proliferation, apoptosis and differentiation. Cell proliferation and migration in various mammalian cells can be inhibited by K^+^ channel blockers, and are initiated by wound healing. In keratinocytes, there are different K^+^ channels that are expressed, such as ATP-sensitive, two-pore domain and Ca^2+^-independent/dependent K^+^ channels. Despite this, the modulation and function of these channels are still not fully understood [28,29,30].

The transient receptor potential (TRP) channels are deeply involved in the mechanical and electrical processes of wound healing.

TRP proteins, which are functional channels, are crucial for cellular ion homeostasis; they are primarily Ca^2+^ and Na^+^ channels. Different cell types have various TRP channels which are expressed in abundance. Examples of cells that express TRP channels are keratinocytes, melanocytes, fibroblasts and various immune cells [38,39].

The activation of TRP channels may be triggered by external stimuli or local environmental changes including pain, pruritus, heat, warmth or cold, odor, mechanical stimulation and osmotic pressure changes [40]. Additionally, TRP channels play an important role in physiological processes like regulating skin homeostasis, melanin synthesis, wound healing and epigenetic regulation. Ultraviolet radiation can cause pathological processes such as barrier damage, vascular stress relaxation, oxidative stress and skin cancer.

The TRP family is emerging as a key player in the regulation of fibrosis in different diseases. At present, 28 different mammalian TRP channels have been identified, comprising six TRP families (TRPA (for ankyrin with one isoform), TRPC (“canonical”, for classical with seven isoforms), TRPM (for melastatin with eight isoforms), TRPML (for mucolipidin with three isoforms), TRPV (for vanilloid with six isoforms), TRPP (for polycystin with three isoforms)) [41,42].

TRP proteins are composed of intracellular N- and C-termini, six-membrane-spanning helices (S1–S6) and a presumed pore-forming loop (P) between S5 and S6. In their C-terminal tails, most members of the TRP family exhibit an invariant sequence known as the TRP box, which contains the amino acid sequence EWKFAR, and ankyrin repeats at their N-termini. To make a functional TRP ion channel complex, it is necessary to have four monomers that are either homotetrameric or four different TRP monomers that form a heterotetrameric channel [41,43].

The reason why these subfamilies have little in common is that they were created based on sequence homology rather than function. For example, TRPM2 is a redox sensor in macrophages; TRPM7 provides a major Mg^2+^ uptake pathway in intestinal epithelial cells; and TRPM8 detects cold and menthol in sensory neurons, but also regulates epithelial growth and metastasis in response to androgens in the prostate [44,45].

Up to 18 ankyrin repeats with a presumed location in the cytoplasm are found on the TRPA1 protein, which are followed by the six-membrane-spanning- and single-pore-loop-domains that constitute all TRPs. Nociception is influenced by TRPA1 activation of sensory neurons [46].

Members of the TRPC subfamily [47,48,49,50] fall into the subgroups outlined below. Humans have TRPC2 as a pseudogene. The majority of TRPC channels, including the Gq/11-coupled receptors and receptor tyrosine kinases, are believed to be activated downstream of these receptors [51]. The association of TRPC channel hetero-oligomeric complexes with proteins leads to the formation of signaling complexes. It has been suggested that TRPC channels act as store-operated channels (SOCs) (or components of multimeric complexes that form SOCs) that are activated by a decrease in intracellular calcium stores [19,52,53]. The evidence suggests that conventional store-operated mechanisms are not used to directly gate them, unlike Stim-gated Orai channels. In physiologically relevant ranges of force, TRPC channels do not have a mechanical gate [54].

TRPM1-8 is the largest subfamily of TRP channels, consisting of eight members, and has a different structure and physical function among the TRP channels. In common with other TRP channels, TRPMs have cytoplasmic N- and C-terminals separated by six putative transmembrane (TM) domains with the pore-forming region found in the loop between TM5 and TM6; the TRPM4 selectivity filter is also located in this region [55]. TM4 and the TM4–TM5 linker in TRPM8 determines its sensitivity to voltage, temperature and chemicals, i.e., menthol [56], while the distal part of TM6 determines cation versus anion selectivity, at least in TRPM2 and TRPM8 channels [56]. Similarly to TRPC channels, they have a TRP box in the C-terminal. Their N-terminus lacks ankyrin repeats found in TRPCs and TRPVs, but instead has a common large TRPM homology domain. Functional TRP channels are most likely homo- or hetero-tetramers, and the C-terminus spiral domain is necessary for TRPM channel assembly and sufficient for tetrameric formation [57].

TRPA1 activates the inflammatory response in keratinocytes by amplifying the power of inflammatory cytokines and prostaglandin E2 (PGE2), both involved in skin inflammation and pruritus [58]. In allergic skin diseases, the increase in pro-inflammatory cytokines is caused by the stimulation of heat-shock proteins (HSP) growth [59,60]. In summary, keratinocytes produce a variety of inflammatory mediators as a result of TRPA1 activation.

TRPV family members are grouped into non-selective cation channels, TRPV1-4, and more calcium-selective channels, TRPV5 and TRPV6.

TRPV1 is a mammalian TRP channel that is widely characterized. TRPV1 was first cloned from rats with an open reading frame of 2514 nucleotides that encodes a 95-kDa, 838-amino-acid protein. TRPV1 is made up of a long 400-amino-acid amino-terminus that contains three ankyrin-repeat domains and a carboxy-terminus that contains a TRP domain that is close to S6 in structure. Functional TRPV1 channels exist as homo- or heterotetramers (co-assembling with TRPV3) [41,61]. A recent study that combined spectral fluorescence resonance energy transfer (FRET) and single channel measurements showed that all temperature dependent TRPVs (thermoTRPVs) can produce heteromers, and these heteromers have distinctive conductance and gating properties, which may cause a greater functional diversity [41].

This channel generates several cellular signals when activated, including membrane depolarization and a rise in cytoplasmic calcium. Peptidergic peripheral sensory neurons that are involved in the perception of pain have TRPV1 expressed at the highest level. When overexpressed recombinantly in cell lines, the activation of TRPV1 is possible by capsaicin, the primary pungent ingredient in chili peppers, or related chemical compounds that have a vanilloid chemical group, so this is why the subfamily of “transient receptor vanilloids” bear this name. Extracellular protons can cause TRPV1 to become active, while small lipophilic molecules like N-arachidonoyl dopamine and anandamide can also activate this channel [59,60]. Other chemical agonists can also act as agonists, such as 2-aminoethoxydiphenyl borate (2-APB), which has been shown to be a dose-dependent activator and inhibitor of IP3 and store-operated calcium channel (SOCE).

Chemical and thermic stimuli, i.e., capsaicin found in chili peppers and high temperature (>43 °C), affect the TRPV1 channel. TRPV1 is found in skin cells, like keratinocytes, mast cells and dendritic cells, which are responsible for sensing pain and responding to chemical stimuli. TRPV1 activation and rapid Ca^2+^ influx cause neurogenic inflammation by releasing neuropeptides like substance P (SP) and calcitonin gene related peptide (CGRP) [57,62]. The release of neuropeptides and other mediators during cutaneous inflammation of a nerve secondary leads to the activation or sensitization of TRPV1, which maintains cutaneous neurogenic inflammation.

TRPV2 is a non-selective cation channel that is activated by mechanical, chemical and thermic stimuli, i.e., high temperatures, such as >52 °C, cannabidiol, 2-APB, probenecid, and mechanical stress. Under normal conditions, TRPV2 is found in the endoplasmic reticulum in most cells. TRPV2 can be activated by phosphatidylinositol 3-kinase-activating ligands to translocate to the plasma membrane where it is functional as a cation channel [41,61]. It was observed that mechanical stress can lead to the translocation of the TRPV2 channel to the plasma membrane.

The discovery of TRPV3 occurred in 2002 when Peier et al. isolated cDNA from the skin of newborn mice [63]. TRPV3 is a Ca^2+^-permeable nonselective cation channel [60,61,64]. It is currently an insufficiently studied channel that is part of thermosensitive ion channels and detects heat [65]. TRPV3 can be found in keratinocytes, and it is involved in maintaining and functioning of the skin barrier, as well as in promoting the development of inflammatory skin diseases, pain signal transmission, and hair morphogenesis [64,65]. In addition to maintaining Ca^2+^ homeostasis, TRPV3 channels expressed in the rumen (a part of the stomach) are also important for transporting NH_4_^+,^ Na^+^ and K^+^ across the stomach in ruminants [66,67,68,69]. The influx of NH_4_^+^ is stimulated by the expression of human TRPV3 in cells and may play a role in nitrogen metabolism [70].

The initial discovery of TRPV4 was as a TRP channel that is widely expressed, and it can be stimulated by changes in extracellular osmolarity. This channel is activated by multiple physical and chemical stimuli, including cytochrome P450 metabolites of arachidonic acid, and warm temperatures. TRPV4 has been linked to numerous health and disease processes, many of which involve the skin, as demonstrated by its expression pattern and extensive range of activators [61].

The TRPV subfamily includes TRPV5 and TRPV6 channels that have a high level of calcium selectivity. They can form either homo- or heterotetramers. TRPV5 is most abundantly expressed in the kidney, whereas TRPV6 is most abundantly expressed in the intestine. The expression of TRPV5 and TRPV6 was not found in ER, but the ER can indirectly affect their expression at the plasma membrane [71]. The stromal interaction molecules in a helix–loop–helix structural domain or motif found in a large family of calcium-binding proteins (STIM1 EF-hand) domains recognize calcium depletion from the ER. STIM1 will be able to unfold due to this recognition. STIM1 can interact with SOCE in this manner. SOCE channels can induce calcium entry to enable TRPV6 translocation to the plasma membrane by interacting with the Annexin1/S100A11 complex in prostate cancer cells. TRPV5 and TRPV6 are capable of selectively removing calcium and constitutively activating it, which could lead to a large amount of calcium entering the cell and filling up the calcium pool in the ER. The Annexin1/S100A11 complex may vary depending on the tissue. For prostate cancer, Annexin1/S100A11 is present, while Annexin 2/S100A10 complex is present in the intestine and kidney [71,72,73].

The aim of the study was to review the mechanisms of ion channel involvement in wound healing in in vivo experiments in murine (mice, rats) and how can this process be influenced. This review used the latest results published in scientific journals over the last year and this year to date (1 January 2023–31 December 3000) in order to include the in-press articles.

## 2. Topics in Wound Healing

The results of the study are presented in Table 1, Synoptic review of the studies with in vivo protocols of wound healing in murine published in 2023 and 2024 to date.

### 2.1. Wound Healing and Endogenous Electric Fields

The wound healing mechanism is represented by a dynamic cascade of ion channels. Transepithelial potential (TEP) difference is the voltage across an epithelium, and represents a sum of the membrane potentials between the outer and inner cell layers. TEP measurements over time in the wound were corroborated with histological changes and TEP variations during porcine skin wound healing. The expression of a crucial element implicated in Na^+^ transport, Na^+^/K^+^ ATPase pumps, was also evaluated at the same time points during the re-epithelialization process. The ascending and decreasing TEP values were correlated with changes in the expression of these pumps. The distribution of Na^+^/K^+^ ATPase pumps also varied according to epidermal differentiation. Further, inhibition of the pump activity induced a significant decrease in the TEP and of the re-epithelization rate. The transepithelial potential (TEP) present in non-lesional epidermis decreases and induces an endogenous direct current epithelial electric field (EF) that could be implicated in the wound re-epithelialization. Epithelial cells migrate in a directional manner in response to electrical signals, with the majority of them detecting minute EFs at the cathodal pole at the center of the wound. It has been long believed that EFs, which are naturally occurring, aid in wound healing by directing cell migration [32,35,82]. In response to applied EFs, large epithelial sheets of keratinocytes or corneal epithelial cells have shown collective directional migration, but this was only recently demonstrated [32,35,82]. The EFs result from the movement of sodium, potassium and calcium ions to their corresponding ion channels.

In a recent study of our research team, it was demonstrated that blocking potassium channels leads to wound healing. Since the potassium current is a depolarizing current, while the calcium and sodium currents are repolarizing, the blocking potassium current increases the membrane potential, which is counteracted by blocking the calcium and sodium currents [83,84].

Inhibition of the Ba^2+^-insensitive large conductance Ca^2+^-activated K^+^ (BKCa) channels can increase the migration and proliferation of normal human epidermal keratinocytes (NHEKs) during wound healing, according to another study. Accelerating cutaneous wound healing may be possible by down regulating or inhibiting BKCa channels [32,35,85].

### 2.2. TRPC Role in Wound Healing

TRPs are involved in tissue repair processes. It has been demonstrated that human keratinocytes express almost all TRPC channel subtypes, including TRPC1, TRPC3, TRPC4, TRPC5 and TRPC6. Knocking down either TRPC1 or TRPC4 in keratinocytes can decrease SOCE and inhibit keratinocyte differentiation, which makes the functional significance of expression of TRPC1 and TRPC4 evident [86]. TRPC6 has been found to play a key role in the differentiation of human keratinocytes. TRPC6 seems to be involved in the mechanism that promotes karyocyte differentiation, as demonstrated by the small molecules from the triterpene family both in vitro and in vivo [86].

### 2.3. TRPV Role in Wound Healing

The TRPV family, which includes TRPV1, TRPV2, TRPV5 and TRPV6, is highly expressed in neutrophils [55,56]. By increasing inflammation and modifying collagen deposits in the rat model of polycystic ovary syndrome (PCOS), the delay in cutaneous re-epithelization shows the involvement of TRPV1 [87]. Calcium entry dependent neutrophil activation and release of pro-inflammatory cytokines could be a consequence of TRPV1 activation in neutrophils in humans with PCOS [57]. According to another report, TRPV1 is involved in the migration of neutrophils induced by activation of the leukotriene B4 receptor (LTB4) [62]. Keratinocytes are the primary source of TRPV1 activation, which causes cutaneous inflammation and delayed healing in tape strip model, burns and UVB wounds [58]. The strongest evidence of such expression comes from human keratinocytes. TRPV1 mRNA and immunoreactivity similar to TRPV1 were discovered in cultured human keratinocytes. In a recent study TRPV1 antagonist capsazepine prevented the calcium influx in keratinocyte cell cultures which is triggered by capsaicin (TRPV1 agonist) or protons [86].

Furthermore, the loss of TRPV1 suppressed inflammation during the healing of alkali-burned mouse corneas, and TRPV1 blockade was also reported to delay debrided epithelial wound healing in the cornea [78,88,89].

According to another study, mice without TRPV1 had persistent neutrophilic inflammation and determined the formation of neutrophil extracellular TRAPS, which hinders the healing of murine cutaneous wounds in vivo [78].

There is a significant amount of evidence suggesting that TRPV1 may have a direct impact on epidermal biology through its expression in keratinocytes. Cultured human keratinocytes had TRPV1 receptor mRNA and immune reactivity that is similar to TRPV1 in neurons [88]. These keratinocyte cell cultures showed a calcium influx induced by capsaicin or protons, which was blocked by capsazepine.

In a phase 2 clinical trial, topical ocular administration of an agonist of TRPV1, SAF312 2.5% (dosed four times daily for 3 days) demonstrated efficacy in reducing the severity of ocular pain associated with corneal epithelial defect after post–photorefractive keratectomy surgery. The drug was well-tolerated, and there was no delay in wound healing [90].

The presence of TRPV2 is described in macrophages, mast cells, natural killer cells, dendritic cells, lymphocytes, neuroendocrine cells and certain cancer cells. TRPV2 is responsible for regulating multiple cell functions, such as cytokine release, chemotaxis, phagocytosis, endocytosis, inflammasome activity and podosome assembly [38,89,91]. In rosacea there is an increased immunoreactivity in macrophages and mast cells in human skin, similar to that induced by TRPV2 one.

In mammal skin (keratinocytes), particularly in hair follicles and the basal layer of the epidermis, there is a high expression level of TRPV3 [63,66,92,93,94,95]. The expression of TRPV3 is also present in human sensory neurons, spinal motor neurons, and peripheral nerves, but it is not yet known if it is also expressed in rat and mouse sensory neurons [96,97,98].

TRPV3 mRNA and protein are prominently expressed in skin keratinocytes. TRPV3, along with other TRPV channels, can be activated by thermal or chemical stimuli. Reports indicate that 2-APB, Farnesyl Pyrophosphate, and various plant extracts, including camphor, carvacrol, eugenol and thymol, are agonists of TRPV3. TRPV3 responses to agonists stimulation can be enhanced by multiple factors, including unsaturated fatty acids, repetitive heat stimulation or cholesterol. Factor-inhibiting-hypoxia inducible factor has the ability to suppress TRPV3 activity, which is due to oxygen-dependent hydroxylation. TRPV3 has been proven to regulate the release of multiple mediators during heat therapy, which can have an impact on neuronal activity. The co-cultures between keratinocytes and sensory neurons were reported to be activated by heat, that caused a rise in keratinocyte calcium levels, followed by a delayed calcium influx response in the neurons. It is evident that the effect was mediated by ATP signaling and relied on keratinocyte TRPV3. TRPV3 plays a role in the release of nitric oxide in keratinocytes by heat through a mechanism that is unrelated to nitric oxide synthase. A recent study demonstrated that TRPV3 modulation has an impact on both bacterial phagocytosis and bacterial cell clearance by macrophages [74].

Another study confirmed these facts by using two antagonists applied topically on the sore dorsal skin or swollen ear in KO mouse, which resulted in a dose-dependent reduction in inflammation [79].

Skin cells have a high expression of TRPV4. This channel plays a significant role in the maintenance of the epidermal barrier by elevating intracellular calcium levels in human keratinocytes and promoting the formation of cell–cell junctions. TRPV4 expression is decreased by suppressing cultured human keratinocytes [37,92]. The recovery of the epidermal barrier can be quicker with increased temperatures and with chemical agonists of TRPV4, in human skin tissues when stratum corneum was removed. It has been suggested by recent research that TRPV4 may have an impact on fibrosis, angiogenesis, pruritus, mechanical conduction and epigenetic regulation [42,99].

The migration of keratinocytes and fibroblasts, along with the increase in collagen deposition in the wound area, are the outcomes of TRPV4 activation, which conduct to the healing of cutaneous wounds [78].

### 2.4. TRPM Role in Wound Healing

TRPM channels play a significant role in the vasculature, as they are widely expressed in endothelial and vascular smooth muscle cells obtained from various vascular beds. TRPM mRNA expression has been detected by several studies through RNA-PCR analysis, which is the most sensitive and selective technique. All other TRPM genes seem to be expressed in vascular tissues, except for TRPM1 and TRPM5 [100].

According to research, blocking TRPM2 with 2-Aminoethyl diphenylborinate (2-APB) or TRPM2 siRNA can reduce endothelial cells apoptosis and encourage angiogenesis. Consequently, they promote an increase in the integrity of blood–spinal cord barrier and enhance the recovery of locomotor function in rats that have diabetes and spinal cord injury [90].

Another study pointed out that loss of TRPM8 function during wound healing can enhance epithelial repair in the cornea, but it can also increase the risk of epithelial lesions [75].

## 3. Materials and Methods

We performed a large literature scan to emphasize the features of TRP channels. Also, a systematized analysis of the literature on the pharmacological influence of ion channels involved in the process of wound healing in rodents (mice, rats and rabbits) was carried out using tags in the database PubMed (Medline) but limited to 1 January 2023–31 December 3000, due to the fact that this analysis was not performed by other researchers. The tag originally used was “ion channel wound healing”, and resulted in 761 articles for the interval 1940–3000, and for the two years chosen, the search returned 61 results. After reading the full text, we excluded nine articles that did not present the mechanism of action involving ion channels in wound healing. Also, 46 articles presented only in vitro investigations of the process of keratinization or scarring. Only eight articles addressed the in vivo mechanism of the wound healing. Also, we searched inside the references of already reviewed articles. The results are presented in Table 1. An additional 18 articles were also screened.

## 4. Conclusions

The wound healing mechanism is debate for researchers. A hypothesis of the electric-driven wound healing process was issued in 2012. Electric fields and currents seem to appear immediately and spontaneously at injury, grow rapidly, persist for many hours and days during the healing process and disappear immediately at the time of complete healing. It has been shown that this natural, endogenous electrical signal of wounds is a powerful stimulus for cell migration during wound healing, overcoming other factors such as growth factors, chemicals drugs and surface of cutaneous loss.

TRP channels are involved in EF activity, TEP generation and in the activation of immune cells during the inflammatory phase of wound healing. Some TRP channels, such as TRPV1, TRPV3 and TRPA1, are expressed in immune cells and can be activated by inflammatory mediators. It could be stated that the effects of TRP channels on wound healing is significant and can be modified by some drugs. Positive effects on wound healing are obtained by stimulating TRPV1, TRPV4 and TRPA1 using agonists and by inhibiting with antagonists or antisense oligonucleotides, or knocking down TRPV3 and TRPM8.

As it could be seen in Table 2, one could remark that the effects on wound healing of some drugs acting on TRP channels is pharmacological significant.

Comprehension and elucidation of the high cellular diversity, complexity and plasticity of wound healing is a significant challenge. Despite the perplexity of this goal, it is crucial that we strive to fully comprehend the mechanisms that underpin both natural and pathological healing.

Taking into account all the results presented in this paper, one can observe that this subject of wound healing is a very complex one and the influence of ion channels especially TRP ones, is far from being elucidated, more in murine and not at all in human beings. Other limitations might be the lack of data regarding some subtypes of TRP channels, i.e., TRPV5 and TRPV6. Emerging wound models can offer an opportunity to further explore the molecular and cellular aspects of wound repair [7]. The absence of in vivo studies in humans which aim to compare the role of TRP channels in murine wound healing is another limitation, which is why we limited our study to in vivo murine wound healing models.

## Figures and Tables

**Table 1:** Synoptic review of the studies with in vivo protocols of wound healing in murine published in 2023 and 2024 to date.

Study/Reference	Subject Species Animal Used	Strain/Gender	Ion Channel	Substance Agonist/Antagonist	Site of Action	Other Blocking Methods of the Ion Channel	Healing Stage	Limitations of the Study According to Authors	Author’s Conclusions	Our Remarks
Sahu RP et al., 2023 [74]	Mice	Balb/c	TRPV3	FPP (2 μM)-ag/DPTHF (200 μM)-antag	Skin wound healing	TRPV3 SiRNA	Inflammatory	Does not specify the number of mice per batch. The animal gender is not specified.	TRPV3 modulation affects both bacterial phagocytosis as well as bacterial cell clearance by macrophages.	In the settings of infection of the skin, TRPV3-activator treated sample presented a better-healed tissue with more blood vessels there.
Ran L. et al., 2023 [75]	Mice	C57BL/6J and Trpm8−/−	TRPM8	Menthol and Tacrolimus- ag/	Corneal epithelium	KO-of the gene	Proliferative		The cornea’s epithelial repair may be improved by the loss of TRPM8 function in corneal wound healing, but it could also increase the risk of epithelial scars and opacifications.	Loss of TRPM8 function promotes re-epithelization after debridement.
Zhang S. et al., 2023 [76]	Rats	Female/Sprague-Dawley	TRPM2	/2-APB-antag	Spinal cord injury (SCI)	TRPM2 SiRNA	Inflammatory		Inhibition of TRPM2 with 2-APB or TRPM2 siRNA will ameliorate the apoptosis of endothelial cells and promote angiogenesis, subsequently enhance blood-spinal cord barrier integrity and improve the locomotor function recovery of diabetes combined with SCI in rats.	TRPM2: antagonism restore BHE integrity; ↓Reactive oxygen species through suppression of p-CaMKII/eNOS; ↑angiogenesis in some conditions: type 1 diabetes mellitus (T1DM) and SCI.
Ueno K. et al., 2023 [77]	Mice	TRPV1 KO and C57BL/6N/Male and female mice	TRPV1	TRPV1 KO and WT mice	Wound produced by dorsal excision	KO of the gene of TRPV1	Inflammatory		Postoperative day 7 and 10 showed a significant increase in the remaining cutaneous lesion in KO than WT mice. Histological examination revealed a significant delay in re-epithelialization in KO mice at postoperative day 7.	↓ re-epithelization in KO mice; ↑ Neutrophil Extracellular TRAPS in KO mice.
Taivanbat B. et al., 2023 [78]	Mice	WT and TRPV4 KO mice.	TRPV4		Wound produced by dorsal excision	KO of the gene	Inflammatory	Gender and/or the animals’ numbers are not specified	The migration of keratinocytes and fibroblasts and the increase in collagen accumulation in the wound area depended on TRPV4, which promotes cutaneous wounds healing.	On days 2–6, KO mice experienced a delay in healing. On day 4, the WT mice experienced a smaller lesion. KO mice have less granular tissue. The wound healing process is more complex and some intermediary data are conflicting with the final results.
Qu Y. et al., 2023 [79]	Mice	male and female/black C57BL/6 mice	TRPV3	/Osthole and Verbascoside-antag	Ear exhibiting swelling and dermatitis induced by a single exposure of weak UVB radiation	KO of the gene	Inflammatory	Mice of both genders were used. Small number of mice per batch. The numbers of male and female mice are not specified	The TRPV3 gene KO can alleviate UVB-induced ear swelling and dorsal skin inflammation. The inhibitors of TRPV3, osthole and verbascoside, that were topically applied showed a dose-dependent reduction in skin inflammation and lesions.	The TRPV3 gene is up-regulated at the site of UVB irradiation both in fibroblast and keratinocytes and pharmacological manipulation of TRPV3 could alleviate the swelling and inflammation.
Opas I.M. et al., 2023 [80]	Mice	WT and TRPA1-deficient male B6;129P-Trpa1(tm1Kykw)/J mice	TRPA1		Scleroderma like induced by bleomycin	KO of the gene	Inflammatory	Mice of both genders were used. Small number of mice per batch. There is no control for KO mice batch. There were different amounts of solvent DMSO used, which is a very well-known tissue preservative and substance involved in cell survival. The numbers of male and female mice are not specified.	Bleomycin-induced scleroderma is aggravated by acting on TRPA1 which enhances fibrotic and inflammatory responses. TRPA1-blocking therapy has the potential to reduce M2 macrophage-driven diseases (macrophages depending on the exposure to IL4, IL10, IL13) such as systemic sclerosis and scleroderma.	Mice with TRPA1 deficiency had lower expression of collagens 1A1, 1A2 and 3A1 after taking bleomycin and manifest improvements in this model of cutaneous sclerosis.
Choi CR. et al., 2024 [81]	Rats	Sprague Dawley rat male	BKCa	/KCl-antag		BKCa SiRNA in rats	Proliferative	The researchers did not utilize a splint in order to inhibit wound contraction.	Keratinocyte wound healing is accelerated by blocking the BKCa channel with KCl and by influencing cell proliferation, migration and F-actin polymerization.	In the group treated with 25 mM KCl, the wound sizes at 7, 14 and 21 days post-injury were significantly smaller than those in the control group.

Abbreviations: TRPV1—transient receptor potential cation channel subfamily Vanilloid member 1, TRPV3—transient receptor potential cation channel subfamily Vanilloid member 3, TRPV4—transient receptor potential cation channel subfamily Vanilloid member 4, TRPA1—transient receptor potential cation channel subfamily A member 1, TRPM2—transient receptor potential cation channel subfamily Melastatin member 2, TRPM8—transient receptor potential cation channel subfamily Melastatin member 8, BKCa channel—the large-conductance Ca^2+^-activated K^+^ channel, KO—knock-out, WT—wild type, ag—agonist, antag—antagonist, KCl—Potassium chloride, FPP—para-Fluorophenylpiperazine, SiRNA—Small interfering RNA, DMSO—Dimethylsulfoxide, ↓—decreased, ↑—increased.

**Table 2:** The effects of influencing TRP ion channels on wound healing summarized from the reviewed articles.

Study/Reference	Ion Channel	Positive/Negative Influence in Wound Healing
Ueno K [77]	TRPV1	+
Sahu RP [74]	TRPV2	-
Qu Y [79]	TRPV3	-
Taivanbat B [78]	TRPV4	+
Opas I.M [80]	TRPA1	+
Ran L. [75]	TRPM8	-

## Data Availability

Not applicable.
